# First choice of treatment place in the pathways to epileptic care at the outpatient department of University of Gondar Hospital, Northwest Ethiopia: Cross-sectional institutional based study

**DOI:** 10.1371/journal.pone.0181310

**Published:** 2017-08-15

**Authors:** Berhanu Boru Bifftu, Berihun Assefa Dachew, Bewket Tadesse Tiruneh, Wondale Getinet Alemu

**Affiliations:** 1 University of Gondar College of Medicine and Health Science, Department of Nursing, Gondar, Ethiopia; 2 University of Gondar College of Medicine and Health Science, Department of Epidemiology, Gondar, Ethiopia; 3 University of Gondar College of Medicine and Health Science, Department of Psychiatry, Gondar, Ethiopia; University of Rome Tor Vergata, ITALY

## Abstract

**Background:**

Epilepsy treatment gap range from 87% to 98%. In spite of this, there is a gross inadequacy of the availability, accessibility and affordability of Anti-Epileptic Drugs. In countries like Ethiopia, where most populations are less aware about mental health problems, most people seek help for their illness from traditional healers. Thus, the main purpose of this study was to assess the pathways to epilepsy care and associated factors.

**Methods:**

Cross-sectional study design utilized among 409 participants selected by systematic random sampling technique. Pathways to epilepsy care were assessed by using the WHO Pathway Study tool. Multivariable logistic regression was done to identify factors associated with pathways to epileptic care.

**Results:**

Overall, 162 (39.6%) of participants first contacted with modern treatment. Two hundred and forty seven (60.4%) of participants counted traditional healers and religious healers were the most common (47.2%). Being men, attending higher education, urban residence, short duration of illness, social support and age at the onset of illness were factors associated with first contact with modern treatment.

**Conclusion:**

Modern treatment was not the first place of choice for the majority of the respondents. Strengthening awareness creation program about epilepsy and its treatment is highly recommended with special emphases to urban dwellers and less educated people.

## Background

Epilepsy is one of the most common neurological disorders, affecting around 50 million people worldwide. Of these 50 million, majority them (80–85%) live in poor countries [1]. In low- and middle-income countries (LMICs), the median estimate for lifetime prevalence of epilepsy is 15.4 per 1000 people in rural areas and 10.3 per 1000 in urban settings and it is the second reason for consultation and hospitalization [2]. In Ethiopia, epilepsy is a huge problem, with an estimated prevalence of 520/100,000 people in a large-scale, rural, community-based survey [3, 4]. In spite of this, there is a gross inadequacy of the availability, accessibility and affordability of Anti-Epileptic Drugs [2–9]. In countries like Ethiopia, where most populations are less aware of health problems, most people seek help for their illness from traditional healers [3, 10, 11]. Use of traditional medicines (TMs) is widespread in developed and developing countries including Ethiopia. Up to 80% of the population in developing countries uses traditional medicine due to the cultural acceptability of healers, the relatively low cost, difficult access to modern health facilities and their perceived cause of the illness [12, 13]. Like other low and middle income countries, many people in Ethiopia also rely on spiritual remedies [13, 14]. Data on help-seeking confirm that decisions and choices about which practitioner to consult were associated with belief, choice or preference and the structural of health service which result in epilepsy treatment gap [10, 11].

The proportion of people with epilepsy who require treatment but do not receive was known as epilepsy treatment gap. Epilepsy treatment gap has been proposed as a useful parameter to compare access to and quality of care for epilepsy patients across populations [15]. This gap ranges from 10% for developed countries and up to 98% for developing countries [4, 16] and 87% to 98% in Ethiopia [4]. Untreated epilepsy is a critical public health issue, leading to devastating consequences such as social, economical and poor health outcomes [17–21], stigma and higher mortality rate [19–22]. In spite of this treatment gap and concern over the consequences of social, economic and health outcome; very little research attention has been focused in developing countries including Ethiopia on the pathway to epileptic care and determinant factors. Therefore the main purpose of this study was to assess the first choice of treatment place in the pathways to epileptic care and associated with first choice of modern treatment factors.

## Methods

### Study design, area and period

Institutional based cross-sectional study was conducted to assess the first choice of treatment place in the pathways to epileptic care and associated factors among epileptic patients attending University of Gondar Hospital (UoGH), from November to December 30/2015. UoGH was located in Gondar town, 748 kilometers away from the capital city of Ethiopia, Addis Ababa. It serves up to 5 million people in the catchment area.

### Participants

Participants of this study were individuals with epilepsy receiving follow-up care at the outpatient department of University of Gondar Hospital. Single population proportion formula (with 5% margin of error, 95% confidence level and 49.3% proportion) was used to calculate sample size; and it was found to be 422 (including 10% non-response rate). By considering the flow of patients for the last 12 months, the average number of patients per day calculated and finally the included participants were selected by systematic random sampling technique. The inclusion criteria for this study were (i) age ≥ 18 years; (ii) confirmed individuals with a clinical diagnosis of epilepsy by physicians and (iii) on follow up at the University of Gondar Hospital.

### Data collection and instruments

Amharic version of semi-structured interviewer administered questionnaire involving: socio-demographic, onset and duration of illness, and the adapted ‘Pathway Study’ encounter form, questionnaires, which was designed for use in series of studies to understand care-seeking and treatment pathways of patients with mental disorder were utilized. This form was used among similar study subjects in Africa and India [11, 13–15, 23].

### Data collection and analysis

The completed questionnaires were coded and entered into EPI info version 3.5.3 statistical software and then exported to SPSS windows version 20 program for analysis. Descriptive statistics (like frequencies, tables, percentages, means and standard deviation) were used for the presentations of socio-demographic, clinical factors and the pathway encounter form. Binary and multivariable binary logistic regressions with odds ratio of 95% confidence interval was used to assess the association of for socio-demographic, onset and durations of the illness with the first place patient sought for care and classified as modern for those participants who get help first directly from at least one of health facilities such as governmental and privet modern health institutions (hospital, health center, clinic and health post) and traditional for those participants who get help first directly from at least one of facilities such as religious (rituals/practices, herbalists and other traditional healings), and self-care (home remedies). All factors with a p-value <0.2 in the bivariate logistic regression were entered into the multivariate model. From the multivariate model, statistical significance was accepted at the 5% level (p < 0.05).

### Ethical consideration

The study proposal was initially approved by the ethical review board of the University of Gondar. A formal letter of permission was obtained from the hospital and submitted to the epileptic clinic. The information about the study was given to the participants. For those participants who agreed to participate in the study, written informed consent was taken and the interviewed in private room.

## Results

A total of 409 participants participated in this study with a 96.9% response rate.

### Socio-demographic characteristics

The majority of the participants were men 257 (63%). The mean (SD) ages of the participants were 28.63 ± 9.70 years. Around half of the participants were completed primary education, 375 (91.7%) were Orthodox Christian by religion and 247 (60.5%) were married in marital status. Three hundred and eighty eight (94.9%) of the participants were Amhara in Ethnicity. Out of 409 participant, 240 (58.7%) were self-employed and 218 (53.3%) were living in urban areas (Table 1).

**Table 1:** Socio-demographic and clinical characteristics of participants.

Characteristics		Number	Percent
**Sex**	Male	257	62.8
	Female	152	37.2
**Age**	18–24	166	40.7
	25–34	126	30.8
	35–44	87	21.3
	>=45	30	7.3
**Educational status**	Can’t read and write	116	28.4
	Primary	199	48.7
	Secondary	52	12.7
	College and above	42	10.3
**Religion**	Orthodox	375	91.7
	Muslim	34	8.3
**Marital status**	Married	248	60.6
	Single	119	29.1
	Divorced/widowed	42	10.3
**Ethnicity**	Amhara	388	94.9
	Tigre	21	5.1
**Employment**	Government	29	7.1
	Student	66	16.1
	Unemployed	74	18.1
	Self-employed	240	58.7
**Residence**	Rural	191	46.7
	Urban	218	53.3
**Social support**	High	249	60.9
	Moderate	95	23.2
	Low	65	15.9
**Age at onset of disease in year**	<6	18	4.4
	6–11	45	11
	12–17	111	27.1
	18–24	113	27.6
	25–34	76	18.6
	>=35	46	11.2
**Duration of the disease**	<1	41	10
	2–5	144	35.2
	6–10	118	28.9
	>=11	106	25.9

### The first choice of treatment place in the pathway to care and perceptions of the illness

Overall, 162 (39.6%) of the participant first contacted the modern treatment. The mean duration to contact the first treatment setting was 7.09 +/-1.02 weeks. The most common 347 (84.8%) sources of information for the preferred site were family/relatives/friends. The most common reasons for choice of place of first treatment were confident of cure 367 (89.7%). For majority 389 (95.1%) of the participants sudden loss of consciousness was the most common symptoms to visit the first place. Regarding the perception of the illness, the most common explanations given for the causation of the illness was spiritual possession 240 (58.7%), majority 373 (91.2%) of the participants perceived that both rich and poor individuals are susceptibility to the illness, 176 (43%) of the participants perceived the illness as severity and majority 349 (85.3%) believed that the illness can be cured with modern treatment (Table 2).

**Table 2:** Distributions of participant by World Health Organization encounter form of the first contact pathways and perceptions of the illness.

Characteristics		Frequency	Percent
**First contact on pathway**	Self-care /home remedies	6	1.5
	Religious (rituals/holy water)	193	47.2
	Herbalists and other traditional healer	48	11.7
	Modern health institutions (government & private)	51	12.5
	Psychiatric clinic	111	27.1
**Mean duration of contact with the first treatment provider**		7.09 +/-1.02	
**Who insisted/informed to visit this place?**	Self	44	10.8
	Family/relatives/friends	347	84.8
	Religious leader	15	3.7
	Others	3	0.7
**Reasons for choice of place of first visit**	Confidence of cure	367	89.7
	Fear of stigmatization	27	6.6
	Proximity	9	2.2
	Lower cost	3	0.7
	Short waiting time	3	0.7
**Main symptoms/problem caused decision to seek care**	Injury	6	1.5
	Mute	3	0.7
	Fail	3	0.7
	Sudden loss of consciousness	389	95.1
	Stress	3	0.7
	Fear	5	1.2
**Perceived causes of epilepsy**	Sinful act	14	3.4
	Fault	6	1.5
	Have cause	37	9
	Unknown cause	240	58.7
	Angry	24	5.9
	Stress	25	6.1
	God	51	12.5
	Heredity	12	2.9
**Who do you think affects by epilepsy?**	Poor	24	5.9
	Rich	12	2.9
	Both	373	91.2
**Perceived severity of epilepsy/ the illness**	Highly severe	167	40.8
	Moderately severe	176	43
	Less severe	63	15.4
	Not at all severe	3	0.7
**Believe epilepsy can be cured**	Yes	349	85.3
	No	60	14.7

### Factors associated with first contact with modern treatment for help seeking

From the bivariate analysis: age, sex, employment, residence, social support, onset of the illness, and duration of the illness were associated with first contact with modern treatment for help seeking at p-value <0.2 and entered in to multivariate analysis. From the multivariate analysis; women (AOR = 0.22, CI: 0.299, 0.655), college and above educational level (AOR = 1.74, CI: 1.221, 9.323), urban residence (AOR = 2.41, CI: 1.376,4.233), social support [high (AOR = 3.66, CI: 1.858,7.123), moderate (AOR = 2.04, CI: 1.566,9.881)], onset of the illness 25–34 year (AOR = 6.62,CI: 2.374,16.878), duration of illness [< 1 year (AOR = 3.87, CI: 2.345,9.667), 2–5 year (AOR = 7.15, CI: 3.428,11.074), 6–10 year (AOR = 4.22, CI: 4.321,15.940)] were factors statistically significant with first contact with modern treatment at p-value <0.05 (Table 3).

**Table 3:** Factors associated with first choice of treatment for help seeking (bivariate and multivariate) analysis, at UoGH, 2015, (n = 409).

Explanatory variables		Pathways to care		COR (95%ci)	AOR(95%ci)	P-value
		Modern n (%)	traditional n (%)			
**Age**	18–24	49(12)	117(28.6)	0.98(0.432,2.392)		
	25–34	65(15.9)	61(14.9)	2.50(1.171,4.946)		
	35–44	39(9.5)	48(11.7)	1.90(2.217,6.282)		
	>=45	9(2.2)	21(5.1)	1		
**Sex**	Men	123(30.1)	134(32.8)	1	1	
	Women	39(9.5)	113(27.6)	0.38(0.315,0.824)	0.22(0.299,0.655)	**<0.001**
**Education**	Can’t read and write	59(14.4)	57(13.9)	1	1	
	Primary	57(13.9)	142(34.7)	0.39(0.222,0.848)	0.41(0.102,0.514)	0.178
	Secondary	19(4.6)	33(8.1)	0.56(0.341,0.791)	0.50(0.512,0.881)	0.007
	College and above	27(6.6)	15(3.7)	1.74(1.839,3.604)	1.74(1.221,9.323)	**0.004**
**Employment**	Government	12(2.9)	17(4.2)	1.10(0.457,2.625)		
	Private	103(25.2)	137(33.5)	1.17(0.430,2.052)		
	Students	18(4.4)	48(11.7)	0.58(0.253,0.703)		
	Unemployed	29(7.1)	45(11)	1		
**Residential**	Rural	93(16.9)	125(30.6)	1	1	
	Urban	69(16.9)	122(29.8)	0.75(0.383,0.960)	2.41(1.376,4.233)	**0.002**
**Social support**	High	123(30.1)	126(30.8)	2.55(1.403, 4.632)3.	3.66(1.858,7.123)	**0.004**
	Moderate	21(5.1)	74(18.1)	0.74(1.995,5.930)	2.04(1.566,9.881)	**0.001**
	Low	18(4.4)	47(11.5)	1	1	
**Age at onset of the illness in year**	**<18**	44(10.8)	130(31.8)	0.37(0.167,11.001)	0.76(0.966,8.573)	0.054
	**18–24**	47(11.5)	66(16.1)	0.78(0.444,9.310)	0.53(0.823,7.117)	0.078
	**25–34**	49(12)	27(6.6)	1.98(1.113,4.106)	6.62(2.374,16.878)	**0.002**
	**≥35**	22(5.4)	24(5.9)	1	1	
**Duration of the illness in year**	**<1**	26(6.4)	15(3.7)	5.07(2.345,10.967)	3.87(2.345,9.667)	**0.003**
	**2–5**	60(14.7)	84(20.5)	2.09(1.185,4.969)	7.15(3.428,11.074)	**0.001**
	**6–10**	49(12)	69(16.9)	2.08(1.172,5.082)	4.22(4.321,15.940)	**0.002**
	**≥11**	27(6.6)	79(19.3)	1	1	

## Discussion

The main findings of our study revealed around 40% of the study participants’ contacted modern treatment, and associated with being men, attending higher education, urban residence, short duration of illness, social support and age at the onset of illness. Compared to other similar studies, the result of this finding was lower than the study carried out in India, 81.6% [15, 16], 62% [11], Kenya 74% [24] Nepal 51.1% [24]. This may be due the fact that those patients from India, Kenya and Nepal may have better access for different resource like: AED, educated man power and health facility. As evidence from Ethiopia revealed lack of epilepsy service include: inaccessibility of medical services, unavailability of anti-epileptic drugs and educated man power was barrier for the utilizations of modern epileptic treatments [10, 13, 25]. In addition to lack of resource, most of the Ethiopian societies consider epilepsy as psychiatric disorder. For example, the only mental hospital, Amanuel specialized hospital, approximately 25% of the customer were epileptic patients that is why individual with epilepsy shared the treatment gap, stigma and discrimination with individual suffering with mental illness [16, 25]. Even if there is scarce of similar study in Ethiopia among epileptic patients, the result of our finding is similar with studies carried out among psychiatric and epileptic patients [3, 10, 14]. From this it is observed the similarity of shared belief of Ethiopian society as evidenced by majority (58.7%) of our study participants’ explain the cause of the illness was spiritual possession [17].

Regarding the source of information, reason for choice of the first place, common symptoms to visit the first place and susceptibility to the illness; majority (84.8%) were family, (89.7%) were confident, (95.1%) was sudden loss of consciousness and (91.2%) were both rich and poor individuals respectively. This result was consistent with study reported from other studies [15, 23, 26].

Regarding the associated factors, women were around 78% (AOR = 0.22, CI: 0.299, 0.655) less likely contact modern treatment compared to men. This is consistent with other study [15]. This may be due to the socio economic dominance of men and women’s lower participation in decision making. Those participants with educational status of college and above were more than one times (AOR = 1.74, CI: 1.221, 9.323) more likely contacted modern treatment compared to those who cannot read and write. This is due to the fact that those patients with higher education status may have accurate, in-depth information about the illness. Moreover, educated individuals have better understanding of the disorder that help them to prevents misconceptions, and reduces concerns about stigma as evidence supported epilepsy education helps people with epilepsy become self-confident, competent in self-management, aware of their needs, and able to access resources to meet their needs [27, 28].

Those patients participated from urban residence were more than two times (AOR = 2.41, CI: 1.376, 4.233) more likely first contact modern treatment compared to the rural participants. This may be due to the lack of infrastructure, accessibility of true information regarding the misconception about the illness and the dominance of traditional healer. This can be supported by a systematic review confirm that perceptions such as cultural beliefs, stigma, rural residence and distance to health facilities were causes for people with epilepsy to seek treatment from traditional healers [8, 15, 16]. Those participants who have high social support were more than three times (AOR = 3.66, CI: 1.858, 7.123), and moderate social support were two time (AOR = 2.04, CI: 1.566, 9.881) more likely contact modern treatments than those patients who had low social support respectively. This is consistent with other study [15]. This may be due to those individual with high and moderate social support have the chance to get correct information on modern pharmacological and psychological treatment that may help them to correct problems such as perceived stigma, misperception about the illness and facilitate treatment seeking. Those patients with an illness onset of age 25–34 years were more than six times (AOR = 6.62,CI: 6.62(2.374,16.878) more likely contacted modern treatment compared to those patient with age greater than or equal to 35. Those patients with less than one year of illness duration were more than three times (AOR = 3.87, CI: 2.345, 9.667), 2–5 years were more than seven times (AOR = 7.15, CI: (3.428, 11.074) and 6–10 years were more than four times (AOR = 4.22, CI: (4.321, 15.940)] were more likely contact modern treatment compared to those patient with duration of illness greater than or equal to 11 years. This is consistent with other study [28]. This may be due to the fact that individuals’ knowledge or beliefs of the treatment place that means those patients who have correct information about the illness contact modern treatment earlier than those did not have correct information because it may take time to get correct information that help them to decide on the preferred place for help.

## Limitations of the study

Recall and response biases might have occurred since; we relied on respondents’ retrospective recall of information. The other possible limitation might occurred when some of the participants might not truly acknowledge and give true response to their previous source of first contact for care (i.e traditional healer) since some of this settings might not recommended in certain social and religious groups.

## Conclusion and recommendations

Modern treatment was not the first place of choice for the majority of the respondents. Education status, urban residence, social support, onset and duration of illness were factors statistically associated with first contact with modern treatment. Strengthening the educational status of epileptic patients was suggested especially for those patients reside in rural area and do not have social support.

## References

[1] Radha krishnan K. Challenges in the management of epilepsy in resource-poor countries. Nat Rev Neurol. 2009;5:323–30. doi: 10.1038/nrneurol.2009.53 19455183 10.1038/nrneurol.2009.53

[2] Chin. Epilepsy treatment in sub-Saharan Africa: closing the gap. African Health Sciences. 2012;12(2):186–92. doi: 10.4314/ahs.v12i2.17 23056026 10.4314/ahs.v12i2.17 PMC3462534

[3] Tekle-Haimanot R A M, Forsgren L, Gebre-Mariam A, Heijbel J, Holmgren G, Ekstedt J. Attitude of rural people in central Ethiopia towards epilepsy. Soc Sci Med 1991;32(2):203–9. 2014416 10.1016/0277-9536(91)90061-g

[4] Tekle-Haimanot R F L, Ekstedt J. Incidence of epilepsy in rural central Ethiopia. Epilepsia. 1997;38:541–6. 9184599 10.1111/j.1528-1157.1997.tb01138.x

[5] Cortes DE Ra LH. “Help-seeking pathways: a unifying concept in mental health care,”. American Journal of Psychiatry. 1993;150(4):554–61. doi: 10.1176/ajp.150.4.554 8465869 10.1176/ajp.150.4.554

[6] Jacoby A S D, Baker G. Epilepsy and social identity: the stigma of a chronic neurological disorder. Lancet Neurol 2005;4:171–8. doi: 10.1016/S1474-4422(05)01014-8 15721827 10.1016/S1474-4422(05)01014-8

[7] Chisholm D W-C. Cost-effectiveness of first-line antiepileptic drug treatments in the developing world: a population-level analysis. Epilepsia. 2005;46:751–9. doi: 10.1111/j.1528-1167.2005.52704.x 15857443 10.1111/j.1528-1167.2005.52704.x

[8] Meyer AC D T, Ma J, Saxena S, Birbeck G. Global disparities in the epilepsy treatment gap: a systematic review. Bull World Health Organ 2010;88:260–6. doi: 10.2471/BLT.09.064147 20431789 10.2471/BLT.09.064147 PMC2855595

[9] Meyer AC D T, Boscardin WJ, Escarce J, Saxena S, Birbeck GL. Critical determinants of the epilepsy treatment gap: a cross-national analysis in resource-limited settings. Epilepsia. 2012;53(12):1–6. 10.1111/epi.12002 PMC3809906 23106784

[10] Berhanu Shibru A S, Asmera Jilalu and Prevett Martin. Primary care treatment of epilepsy in Rural Ethiopia. EthiopJHealth Dev. 2002;16(3):235–40.

[11] Munthali A, Braathen S.H., Grut L., Kamaleri Y. & Ingstad B. ‘Seeking care for epilepsy and its impacts on households in a rural district in southern Malawi African Journal of Disability. 2013;2(1):8. 10.4102/ajod.v2i1.54 PMC5442585 28729991

[12] Organization WH. Traditional Medicine Strategy Geneva: 2002–2005.

[13] Kebede D, Alemayehu A., Binyam G., & Yunis M. A historical overview of traditional medicine practices and policy in Ethiopia. Ethiopia EthiopJHealth Dev. 2006;20(2):127–34.

[14] Bekele Y. Y. F AJ, Alem A. and Baheretebeb Y.. Pathways to psychiatric care in Ethiopia Psychological Medicine. 2009;39:475–83. doi: 10.1017/S0033291708003929 18606050 10.1017/S0033291708003929

[15] Sinha Abhik Ma S S D. Healthcare-Seeking Behavior of Patients with Epileptic Seizure Disorders Attending a Tertiary Care Hospital, Kolkata. Indian J Community Med. 2012;37(1):25–9. doi: 10.4103/0970-0218.94018 22529536 10.4103/0970-0218.94018 PMC3326803

[16] Pal DK D T, Sengupta S, Chaushury G. Help-seeking patterns for children with epilepsy in rural India: implications for service delivery. Epilepsia 2002;43:904–11. 12181010 10.1046/j.1528-1157.2002.47601.x

[17] Mbuba CK N A, Fegan G, Ibinda F, Muchohi SN, Nyundo C, Odhiambo R, et al Risk factors associated with the epilepsy treatment gap in Kilifi, Kenya: a cross-sectional study. Lancet Neurol 2012;11:688–96. doi: 10.1016/S1474-4422(12)70155-2 22770914 10.1016/S1474-4422(12)70155-2 PMC3404220

[18] K R. Global Campaign against Epilepsy: the treatment gap. Epilepsia 2002;43:31–3. 12190976 10.1046/j.1528-1157.43.s.6.13.x

[19] Meyer AC DT, Ma J, Saxena S, Birbeck G. Global disparities in the epilepsy. Nilsson L. Risk factors for sudden unexpected death in epilepsy: a case control study. Lancet 1999;353:888–93. 10.1016/s0140-6736(98)05114-9 10093982

[20] Bifftu BB D B, Tiruneh BT. Perceived stigma and associated factors among people with epilepsy at Gondar University Hospital, Northwest Ethiopia: a cross-sectional institution based study. Afri Health Sci. 2015;15(4):1211–9. 10.4314/ahs.v15i4.21 PMC4765415 26958023

[21] Bifftu Berhanu Boru D BA, Tiruneh Bewket Tadesse and Birhan Tebeje Nigusie Depression among people with epilepsy in Northwest Ethiopia: a cross-sectional institution based study. BMC Res Notes. 2015;8(585):1–8. 26482788 10.1186/s13104-015-1515-z PMC4617742

[22] Ding D W W, Wu J, Ma G, Dai X, Yang B, Wang T, et al Premature mortality in people with epilepsy in rural China: a prospective study. Lancet Neurol 2006;5:823–7. doi: 10.1016/S1474-4422(06)70528-2 16987728 10.1016/S1474-4422(06)70528-2

[23] Subedi s s p, shakya r, pandey ak. Study of pathway to care among patients with epileps. Journal of universal college of medical sciences. 2013;1(1):1–6.

[24] Mbuba CK N A, Fegan G, Ibinda F, Muchohi SN, Nyundo C, Odhiambo R, et al Risk factors associated with the epilepsy treatment gap in Kilifi, Kenya: a cross-sectional study. Lancet Neurol 2012;11:688–96. doi: 10.1016/S1474-4422(12)70155-2 22770914 10.1016/S1474-4422(12)70155-2 PMC3404220

[25] Ministry of Health. NATIONAL MENTAL HEALTH STRATEGY 2012/13–2015/16, Federal Democratic Republic of Ethiopia Ministry of Health. NATIONAL MENTAL HEALTH STRATEGY 2012/13–2015/16. 2012/13.

[26] Jantzen S, Muller-Godeffroy E., Hallfahrt-Krisl T., Aksu F., Pust B., Kohl B., A. et al A training programme for children and adolescents with epilepsy, and their parents. Seizure. 2009;18(7):478–86. doi: 10.1016/j.seizure.2009.04.007 19477662 10.1016/j.seizure.2009.04.007

[27] Aliasgharpour Mansooreh DN N, Yadegary Mohammad Ali, Haghani Hamid Effects of an educational program on self-management in patients with epilepsy. Elsevier journals of Seizure. 2013;22:48–52. 10.1016/j.seizure.2012.10.005 23122512

[28] Matonda Ma Nzuzi TMMN Thierry, Gilbert Mananga Lelo, Magloire Mpembi Nkosi, Madinga Joule, Constantin Kabwe Kola. Are the children with epilepsy treated traditionally a disadvantaged group? A pilot study. Pan African Medical Journal 2016; 23:229. 2016;23:229.

